# The G7 presidency and universal health coverage, Japan’s contribution

**DOI:** 10.2471/BLT.17.200402

**Published:** 2018-03-19

**Authors:** Haruka Sakamoto, Satoshi Ezoe, Kotono Hara, Eiji Hinoshita, Yui Sekitani, Keishi Abe, Haruhiko Inada, Takuma Kato, Kenichi Komada, Masami Miyakawa, Hiroyuki Yamaya, Naoko Yamamoto, Sarah Krull Abe, Kenji Shibuya

**Affiliations:** a Department of Global Health Policy, Medical Building No. 3, Hongo Campus, University of Tokyo,7-3-1, Hongo, Bunkyo-ku, Tokyo, Japan.; b Ministry of Health, Labour and Welfare, Tokyo, Japan.; c Ministry of Foreign Affairs, Tokyo, Japan.; d National Institute of Infectious Disease, Tokyo, Japan.

## Abstract

**Problem:**

If universal health coverage (UHC) is to be achieved globally, it needs sustained promotion and political awareness and support.

**Approach:**

During its presidency of the Group of Seven (G7) industrialized nations in 2016, Japan aimed to raise the issue of UHC to the top of the global health agenda.

**Local setting:**

Japan has promoted a health agenda at all of the G7 summits since 2000 that it has hosted. Human security has been the core foundation of Japan’s foreign diplomacy for several decades and, consequently, there was no apparent opposition within Japan to the inclusion of UHC on the agenda of the summit in 2016. Other G7 governments appeared keen to promote such coverage.

**Relevant changes:**

Since the 2016 summit, UHC has remained a central agenda item for the United Nations and World Health Organization, even though the leaders of both these global organizations have changed. In 2017, Japan hosted the UHC Forum in Tokyo. The participants, who were the heads of United Nations agencies, politicians and other decision-makers from all over the world, showed their continued commitment towards UHC.

**Lessons learnt:**

In the raising of awareness of an item on the global health agenda, high-level champions are critical. Although they may be very diverse, all relevant stakeholders need to be connected and allowed to discuss policies with each other. Having too many allies can, however, lead to policy fragmentation, especially when there is commitment from the highest echelons within each country.

## Résumé

**Problème:**

Une promotion durable ainsi qu'une prise de conscience et un soutien politiques sont indispensables pour parvenir à une couverture sanitaire universelle à l'échelle mondiale.

**Approche:**

En 2016, dans le cadre de sa présidence du Groupe des sept pays les plus industrialisés (G7), le Japon s'est proposé de mettre la question de la couverture sanitaire universelle au premier plan du programme mondial d'action sanitaire.

**Environnement local:**

Depuis 2000, le Japon assure la promotion d'un programme d'action sanitaire lors de chacun des sommets du G7 qu'il accueille. La sécurité humaine constituant le principal fondement de la diplomatie internationale du Japon depuis plusieurs décennies, il n'y eu aucune opposition apparente au sein du pays à l'égard de l'inclusion de la question de la couverture sanitaire universelle dans l'ordre du jour du sommet de 2016. D'autres gouvernements de pays membres du G7 se sont montrés désireux de promouvoir cette question.

**Changements significatifs:**

Depuis le sommet de 2016, la couverture sanitaire universelle est un point central de l'ordre du jour pour l'Organisation des Nations Unies et l'Organisation mondiale de la Santé, malgré un changement de dirigeants au sein de ces deux organisations mondiales. En 2017, le Japon a accueilli le Forum de la couverture sanitaire universelle à Tokyo. Les participants, à savoir les responsables d'organismes des Nations Unies, des responsables politiques et d'autres décideurs du monde entier, ont affirmé leur engagement durable à l'égard de la couverture sanitaire universelle.

**Leçons tirées:**

Des défenseurs de haut vol sont essentiels pour sensibiliser à l’importance d’une question du programme mondial d'action sanitaire. Malgré leurs éventuelles différences, il est fondamental que toutes les parties prenantes soient liées et puissent discuter ensemble de la politique à mener. Un nombre trop important de défenseurs peut néanmoins entraîner une fragmentation politique, en particulier lorsque l'engagement provient des échelons les plus élevés au sein de chaque pays.

## Resumen

**Situación:**

Si se pretende lograr una cobertura sanitaria universal (CSU) en el mundo, se necesitan la promoción sostenida, y la conciencia y el apoyo políticos.

**Enfoque:**

Durante su presidencia en 2016 del Grupo de los Siete (G7) formado por las 7 naciones industrializadas, Japón se propuso elevar el tema de la CSU hasta el primer puesto de la agenda global de salud.

**Marco regional:**

Japón ha promovido una agenda de salud en todas las cumbres del G7 que ha celebrado desde el año 2000. La seguridad humana ha sido el fundamento de la diplomacia internacional de Japón desde hace ya varias décadas y, en consecuencia, no hubo oposición aparente dentro de Japón para la inclusión de la CSU en la agenda de la cumbre de 2016. Otros estados miembros del G7 parecieron entusiastas para promover esta cobertura.

**Cambios importantes:**

Desde la cumbre de 2016, la CSU ha permanecido como un punto central de la agenda para las Naciones Unidas y la Organización Mundial de la Salud, incluso después del cambio de los líderes de estas dos organizaciones. En 2017, Japón fue sede del Foro de CSU en Tokio. Los participantes, integrados por jefes de las agencias de las Naciones Unidas, políticos y otros tomadores de decisiones de todo el mundo, mostraron su compromiso ininterrumpido con la CSU.

**Lecciones aprendidas:**

Para elevar la conciencia de un punto de la agenda global de salud, son fundamentales los defensores de alto nivel. Aunque pueden ser muy diversas, todas las partes interesadas importantes necesitan estar vinculadas y autorizadas para debatir políticas entre ellas. Sin embargo, tener demasiados aliados puede conducir a la fragmentación de la política, especialmente cuando existe el compromiso de los rangos más altos de cada país.

## ملخص

**المشكلة:**

إذا كان لابد من تحقيق التغطية الصحية الشاملة، فالأمر بحاجة إلى الترويج المستدام بالإضافة إلى الدعم والوعي السياسي.

**الأسلوب:**

خلال رئاسة اليابان لمجموعة الدول الصناعية السبع سنة 2016، فقد عمدت إلى طرح قضية التغطية الصحية الشاملة على قمة جدول أعمال الصحة العالمية.

**المواقع المحلية:**

عملت اليابان على الترويج لجدول الأعمال الصحية في جميع مؤتمرات قمة الدول السبعة التي استضافتها منذ عام 2000. وبحكم تركيز اليابان على الأمن البشري كدعامة أساسية لدبلوماسيتها الخارجية على مدار العديد من العقود، فلم تكن هناك معارضة بادية من قبل اليابان لتضمين التغطية الصحية الشاملة في مؤتمر القمة سنة 2016. وعلى الجانب الآخر، بدت حكومات مجموعة الدول السبع الأخرى حريصة على تعزيز مثل تلك التغطية.

**التغيرات ذات الصلة:**

منذ انعقاد مؤتمر القمة سنة 2016، ظلت التغطية الصحية الشاملة بندًا رئيسًا في جدول أعمال الأمم المتحدة ومنظمة الصحة العالمية، حتى مع التغييرات التي طرأت على رئاسة هاتين المنظمتين العالميتين. وفي 2017، استضافت اليابان منتدى التغطية الصحية الشاملة في طوكيو. أبدى المشاركون – من رؤساء وكالات الأمم المتحدة والسياسيين وغيرهم من صانعي القرار من جميع أنحاء العالم – التزامهم المستمر نحو التغطية الصحية الشاملة.

**الدروس المستفادة:**

في إطار جهود رفع الوعي بأحد بنود جدول الأعمال الصحة العالمية، فمن الحتمي اكتساب التأكيد من جانب جهات رفيعة المستوى. وعلى الرغم من التباين الكبير بين الجهات المعنية، فهناك ضرورة للتواصل بين جميع تلك الجهات، والسماح لها بمناقشة السياسات مع بعضها البعض. ولكن وجود العديد من الجهات المؤيدة قد يؤدي إلى تجزؤ السياسات، وخاصةً عند وجود التزام من المستويات العليا داخل كل دولة.

## 摘要

**问题:**

如果要在全球范围内实现全民健康覆盖 (UHC)，需持续推进工作、提高政治意识，并获得政策支持。

**方法:**

在 2016 年担任七国集团轮值主席国期间，日本致力于将全民健康覆盖 (UHC) 问题提到全球医疗卫生发展议程的首要位置。

**当地状况:**

自 2000 年以来，日本在历届七国集团 (G7) 峰会期间均提出了医疗卫生议程。数十年来，人类安全问题一直是日本外交政策的核心基石。因此，将全民健康覆盖 (UHC) 列入 2016 年七国集团峰会议程时，日本国内并未出现明显的反对声音。其他七国集团 (G7) 成员国也积极促进此项事业的发展。

**相关变化:**

自 2016 年七国集团峰会以来，尽管联合国和世界卫生组织的领导者不断更迭，但全民健康覆盖 (UHC) 始终是这两大全球性组织的核心议程。2017 年，日本在东京举办了全民健康覆盖 (UHC) 论坛。来自世界各地的与会者（联合国有关机构领导者、政客以及其他决策制定者）展现了愿意继续致力于全民健康覆盖 (UHC) 事业发展的决心。

**经验教训:**

高水准捍卫者对提高全球医疗卫生议程的认识至关重要。尽管捍卫者的组织形式可能不尽相同，但所有的利益相关者都需要联合起来、彼此交流、协商策略。但是，成员过多也可能会造成政策分裂，尤其在各国最高阶层参与的情况下

## Резюме

**Проблема:**

Для обеспечения всеобщего охвата медико-санитарными услугами на глобальном уровне необходимо постоянное содействие, политическая осведомленность и поддержка.

**Подход:**

В период своего председательства в группе промышленно развитых стран «Большой семерки» (G7) в 2016 году Япония поставила перед собой задачу поднять вопрос о всеобщем охвате медико-санитарными услугами на первое место в глобальной повестке дня в области здравоохранения.

**Местные условия:**

Япония продвигала повестку дня в области здравоохранения на всех саммитах «Большой семерки», которые она проводила c 2000 года. В течение нескольких десятилетий безопасность человека была основой внешней политики и дипломатии Японии, и, следовательно, в Японии не было явного противодействия включению всеобщего охвата медико-санитарными услугами в повестку дня саммита в 2016 году. Другие правительства стран «Большой семерки» оказались также заинтересованы в содействии такому охвату.

**Осуществленные перемены:**

Начиная с саммита 2016 года всеобщий охват медико-санитарными услугами остается центральным пунктом повестки дня для Организации Объединенных Наций и Всемирной организации здравоохранения, несмотря на то что сменились лидеры обеих этих глобальных организаций. В 2017 году Япония провела в Токио Форум по вопросам всеобщего охвата медико-санитарными услугами. Участники, будучи руководителями представительств Организации Объединенных Наций, политиками и другими ответственными лицами, принимающими решения, со всего мира, продемонстрировали свою неизменную приверженность вопросам всеобщего охвата медико-санитарными услугами.

**Выводы:**

В повышении осведомленности о пункте глобальной повестки дня в области здравоохранения важнейшее значение имеют лидеры высокого уровня. Все соответствующие заинтересованные стороны должны быть объединены в этом вопросе, несмотря на различие мнений, и должны иметь возможность обсуждать политику друг с другом. Тем не менее слишком большое количество союзников может привести к разрозненности мнений в политике, особенно когда в каждой стране прослеживается вовлеченность высших эшелонов в решение глобальных вопросов.

## Introduction

According to the World Health Organization (WHO), universal health coverage (UHC) “means that all people and communities can use the promotive, preventive, curative, rehabilitative and palliative health services they need, of sufficient quality to be effective, while also ensuring that the use of these services does not expose the user to financial hardship.”^1^ One main aim of the Agenda for Sustainable Development, as adopted at the United Nations (UN) General Assembly in 2015, is to achieve such coverage by 2030.^2^

For several decades, the Japanese government has prioritized global health in its international diplomacy. For example, in all of the summits of the Group of Seven (G7) industrialized nations (Canada, France, Germany, Italy, Japan, United Kingdom of Great Britain and Northern Ireland and the United States of America) that Japan has hosted, global health has been a main item on the agenda. When Japan last held the G7 presidency, in 2016, much of its focus was on global health and, particularly, on UHC. In the promotion of UHC to the top of the political agenda at a global level, the experience that the Japanese government gained in 2016, in terms of global health diplomacy, could serve as a useful cornerstone.

In April–June 2016, we conducted interviews with employees of the Japanese ministries of finance, foreign affairs and health, labour and welfare who worked in departments connected to global health. Our aims were to investigate the preparatory process behind the 2016 G7 summit, which was held in the Ise-Shima area of Japan on 26–27 May 2016, and its related meetings, investigate how the Japanese government obtained consensus among the diverse stakeholders involved in the summit and determine which people and which other factors appeared most important in the global promotion of UHC.

## Local setting

By 2016 there was already some precedent for Japan’s prioritization of UHC. For several decades, Japan had promoted health systems strengthening, in conjunction with the idea of human security, as a central tenet of its foreign policy. For example, such strengthening was placed high on the agenda of the G8 summit in 2008, which took place in Toyako.^3^ Within Japan, the political will to include UHC as a top priority within the G7 agenda was already present during preparations for the 2016 G7 summit in Ise-Shima and the 2016 G7 health ministers’ meeting in Kobe. At the same time, UHC was already central to the global health agenda, as clearly indicated in the sustainable development goals.^2^ There was no apparent opposition, from other G7 governments, to the inclusion of UHC on the main agenda of the 2016 G7 summit in Japan. In collaboration with WHO and under the leadership of Chancellor Angela Merkel, the German government, in particular, had already made considerable efforts to promote and support UHC. Responding to the need for health systems strengthening, as indicated in the conclusions of the 2015 G7 summit, which was held in the German village of Krün,^4^ Germany began to develop a roadmap towards UHC in 2015.^5^ After the 2016 G7 summit, Germany hosted the first meeting of G20 health ministers and this resulted in the so-called Berlin Declaration, which indicated the health ministers’ continuous support for UHC.^6^

## Approach

In the preparations for the 2016 G7 summit, the strongest drivers for Japan’s prioritization of UHC appeared to be three high-level Japanese champions of global health: Prime Minister Shinzo Abe; Yasuhisa Shiozaki, a former Minister of Health, Labour and Welfare; and Professor Keizo Takemi, a member of the House of Councillors.

In 2013, Shinzo Abe had made health one of the main pillars of his new strategy to promote Japan’s economic growth^7^ and expressed his interest in global health in general and the global achievement of UHC in particular.^8^ In 2015, he published an article in *The Lancet* – entitled “Japan’s vision for a peaceful and healthier world”, in which he explained how Japan’s priorities, as holders of the G7 presidency in 2016, would include UHC.^9^ Though it remains unusual for a head of state to summarize their political priorities via a medical journal, this article helped demonstrate the Japanese government’s unwavering commitment to the support of UHC globally. Prime Minister Abe also raised the issue of UHC, as an agenda item, in bilateral meetings with several other heads of state and, ultimately, initiated dialogue with G7 governments to cultivate the wider support that enabled Japan to prioritize UHC on the agendas of the 2016 G7 summit and its related meetings.

By 2016, Yasuhisa Shiozaki recognized the main strengths of Japan’s health system and the need to communicate more widely with the global community, in an era of globalization. In conveying strong political messages to the global community at several international conferences, he has been a vocal advocate for global health and UHC.^10^ Like Prime Minister Abe, he has published articles in internationally recognized journals while maintaining dialogues, on UHC, with health ministers and heads of international organizations.

Keizo Takemi has drawn on his academic and policy-making background and published papers that appear to have influenced the advisory processes associated with the 2016 G7 summit. He led and coordinated domestic negotiations for the health agenda at the summit while hosting several meetings with relevant ministry officials.

In 2015–2016, Japan hosted several G7 preparatory committee meetings. At these meetings, Japan chaired dialogues, with other G7 nations, that led to the drafting of the main outcome documents of the 2016 G7 summit and related meetings, i.e. the G7 Ise-Shima Declaration^4^ and the G7 Kobe Communiqué,^11^ and the identification of points of consensus. Over the same period, Japan hosted a series of UHC-relevant conferences: a side event to the Seventieth UN General Assembly in 2015; an international conference on UHC in Tokyo in 2015; and with Germany as a co-host, a side event at the Sixty-ninth World Health Assembly in 2016. At these conferences, Japan used the outcomes of earlier G7 summits as launch-pads for UHC-focused discussions with the representatives of G7 and other governments.

The Sixth Tokyo International Conference on African Development, held in August 2016, was the first such conference to make health a major agenda item.^12^ At the 2016 conference, Yasuhisa Shiozaki and the then President of the World Bank Group, Dr Jim Yong Kim, co-chaired a thematic session entitled “Promoting resilient health systems for quality of life.” In the subsequent negotiations on the conference outcomes, Yasuhisa Shiozaki and relevant ministry officials led the debate, among the representatives of many African countries and international organizations, that ultimately contributed to the so-called Nairobi Declaration and Nairobi Implementation Plan and the outlines of a framework for interventions to support UHC in Africa.^13^^,^^14^ By hosting such high-level events, Japan deepened the UHC debate both within and outside of the G7.^11^ The resultant outcome documents, which are widely distributed and read, serve to promote the UHC agenda globally.

## Relevant changes

Although the G7 summit in 2016 encouraged the global community’s continued commitment to UHC, that commitment may have been weakened when, in the same year, the UN’s Secretary-General, WHO’s Director-General and several other strong advocates for UHC were replaced. At the end of 2017, however, the participants at the UHC Forum in Tokyo, who included the new Secretary-General, new Director-General and high-level politicians from all over the world, professed their sustained support for UHC.

Changes in financial trends that supported UHC were observed. Historically, UHC and health systems strengthening have been under-funded and most donor funding has gone to vertical programmes, such as those directed at human immunodeficiency virus. In 2016, however, there was a transition in which some organizations, such as the Global Fund to Fight AIDS, Tuberculosis and Malaria, began to invest in health systems strengthening and UHC. The Global Fund, together with the World Bank Group, announced that it would contribute 24 billion United States dollars (US$) to those African countries that attempted to achieve UHC by using the framework developed at a side event to the Sixth Tokyo International Conference on African Development.^15^ Throughout 2016, when it held the G7 presidency, Japan committed US$ 1.1 billion to global health institutions.^16^ This financial support demonstrated Japan’s strong political commitment to addressing the global health challenges highlighted at the 2016 G7 summit.

## Lessons learnt

The main lessons learnt from the G7-based Japanese promotion of UHC are summarized in Box 1.Strong leadership can push issues to the top of the political agenda very effectively^17^ and the hosting of high-level political dialogue, both within and outside of G7, can be a very strong driver in promoting a policy agenda. The outcomes of the 2016 G7 summit and related meetings in Japan^4^^,^^11^ are expected to be the basis for future policy-making. Although G7 is an influential body with respect to global health, it cannot raise awareness and move forward the global health agenda optimally without the support of other stakeholders and expansion of the debate beyond G7. In 2019, the G20 summit and the UN high-level meeting on UHC should provide further opportunities for promoting UHC at scale.

Box 1 **Summary of main lessons learnt**

- In the raising of awareness of an item on the global health agenda, high-level champions are critical.
- Although they may be very diverse, all relevant stakeholders need to be connected and allowed to discuss policies with each other.
- Having too many allies can lead to policy fragmentation, especially when there is commitment from the highest echelons within each country.

One remaining potential issue is that, in the global promotion of UHC, Japan has had several powerful allies and no obvious vocal opponents. It has been suggested that too many allies can be detrimental and lead to policy fragmentation.^18^ Governments may disagree over the control and development of global policy. Although the UHC2030 platform was launched as an international framework to coordinate the efforts, by relevant stakeholders and various initiatives, to develop UHC globally, the coordination is still a work-in-progress.^19^ Ultimately, however, UHC2030 is expected to catalyse various initiatives and leverage the expertise of all relevant stakeholders.
